# Applications of Game Theory and Advanced Machine Learning Methods for Adaptive Cyberdefense Strategies in the Digital Music Industry

**DOI:** 10.1155/2022/2266171

**Published:** 2022-06-17

**Authors:** Jing Jing

**Affiliations:** Music Teaching Department, Zhengzhou Preschool Education College, Zhengzhou 450000, China

## Abstract

As the likelihood and impact of cyber-attacks continue to grow, organizations realize the need to invest in specialized methods to protect their digital data and the information they circulate or manage. Due to its broad use, game theory has evolved into a concept that can be applied practically while analyzing and modifying existing cyber protection methods to arrive at the best possible conclusions. This study presents an innovative hybrid model that combines game theory and advanced machine learning methods for adaptive cyber defense strategies. Specifically, a repetitive game methodology is implemented to analyze cyber-attacks and model behaviors and study how defenders and attackers make decisions in a competing field. Based on Bayesian inference, the proposed method can predict the next steps in the game to produce the appropriate countermeasures and implement the best cyber defense strategies that govern an organization. The suggested system introduced to the academic literature for the first time was successfully tested in a particular application scenario involving the digital music industry and coping with impending cyber-attacks.

## 1. Introduction

As the cyberspace landscape evolves rapidly, a dynamic framework emerges in which very delicate balances are observed to make optimal decisions. The asset, information, and data values that modern organizations must manage constantly increase. New cyber-attack techniques are continually developing while existing technological defense systems age, making active defense strategies resilient. Furthermore, economic changes, institutional reorganizations, consumer trends, and legal and regulatory compliance requirements all impact decision-making and the overall development of sound defense strategies for an organization or company. To maintain its competitive advantage, the organization in question will need to continuously improve its defense strategies, which will be based on ongoing knowledge and utilization of the cyber threat landscape [1]. An accurate inventory of all assets classified by their value to the organization is critical in assessing the severity of the risks they face and, as a result, the decisions that must be made about them.

Game theory [2] can be used in cybersecurity to create tangible solutions that will allow the maximum utilization of existing strategies and optimize them to create a robust and long-term security environment at the organization level [3–5]. Using game theory principles, cyber security professionals can implement a network of controls that specialize and, as a result, reduce the risk to their valuable assets. They can also apply areas with a low level of risk, maximizing their return on investment. As a result, using specialized scenarios based on game theory, it is possible to predict the attackers' strategy at each stage of the attack cycle, assisting in developing intelligent models to improve cyber security and the development of new intelligent systems to deter attackers.

This study presents applications of game theory and advanced machine learning methods for adaptive cyber defense strategies in the digital music industry. An innovative application of a repetitive game methodology for cyber-attack analysis is proposed; using Bayesian inference [6], the next steps in the game can be predicted so that the best cyber defense strategies can be implemented to shield an organization from cyber-attacks.

## 2. Literature Review

The literature on game theory for adaptive cyber defense approaches is extensive, whether at the network level, where we must deal with massive amounts of raw data or at the strategy level.

In 2015, Laszka et al. [7] developed a strategy for reducing spear-phishing assaults by targeted per-user filtering criteria. They framed the task of screening harmful e-mails, both targeted and untargeted, as a security game. These defined optimum filtering techniques and demonstrated how they might be computed in practice. They put their theoretical hypotheses to the test by comparing them to two datasets taken from the actual world. Using two different sets of real data, they demonstrated that the recommended baselines result in less harm than the nonstrategic restrictions. In addition, they found that the improvement over nonstrategic criteria was more substantial for the comprehensive information. This improvement was unaffected by an increase in the number of targeted customers. This indicated that their technique scaled effectively, both analytically and in terms of how well it performed.

Schlenker et al. [8] explored the critical issue of allocating cyber alarms to a restricted number of professionals in cyber security activities. They proposed the cyber-alert allocation game to investigate this issue and demonstrated how to compute the defender's best options. They offered a unique technique for addressing implement ability concerns in determining the defender's best marginal tactic to resolve this game. Finally, they presented heuristics for solving big games like the one described and an objective assessment of the suggested framework and solution methods.

Nguyen et al. [9] conducted a study of deep reinforcement learning (DRL) techniques used in cyber protection. They discussed various critical topics, such as DRL-based security approaches for cyber-physical infrastructure, independent intrusion detection approaches, and multiagent DRL-based game theory simulations for cyber-attack defensive tactics. Additionally, extensive debates and potential study paths on cyber security focusing on DRLs are provided. They hoped that this exhaustive assessment would provide the groundwork for and support future research into the ability to develop DRL to address more sophisticated digital privacy concerns.

Alpcan and Basar [10], in 2010, in their book, about network security and game theoretic approaches, aimed to present a theoretical foundation for making resource allocation decisions that balance available capabilities and perceived security risks in a principled manner. They focused on analytical models based on game, information, communication, optimization, decision, and control theories applied to diverse security topics. At the same time, connections between theoretical models and real-world security problems are highlighted to establish the critical feedback loop between theory and practice.

Hemberg et al. [11] presented an architecture for adversarial AI called RIVALS that abstractly replicated the hostile, competing for a coevolutionary mechanism in security contexts. The purpose was to develop a system capable of pro-active cyber security against dynamic automated attackers. They reviewed its present uses and how it is used to develop defensive measures. Further work will involve expanding it to enable more cyber defense purposes, creating more effective or genuine reality methods, and applying other Nash equilibrium-finding algorithms to other cyber security challenges with established Nash equilibria [12] and analyzing efficiency.

## 3. Proposed Game Strategy

Many cyber-attacks follow a pattern built on repeating tactics or procedures over time. Infrastructure vulnerability control strategies, for example, can compete with current defensive systems and change their applications over time. In this sense, attackers and defenders engage regularly, and these interactions may be depicted using repetitive games, which are a type of dynamic game [13]. The concept described here is using a repeated game to evaluate cyber-attacks and simulating some cooperative behaviors without a clear endpoint. A repetitive game begins with a static game repeated infinitely or intermittently many times. A reward is given to each player who completes a specific action in this strategic stage game. The sum of each player's gains throughout the game constitutes their final reward. In addition, Bayesian inference [14] can predict the next steps in the game to produce the appropriate countermeasures and implement the best cyber defense strategies that govern a complex system with high uncertainty [15].

The starting point for modeling the proposed system is a static game of the following format:

(1) $$\begin{array}{c} \Gamma =\left\{N,{\left(X_{i},u_{i}\right)}_{i\in N}\right\}, \end{array}$$

where Ν = {1, 2,. .., *n*} is the set of players, *X*_*i*_ is the set of the player's *ί* pure strategies, and *u* is its performance function. Assume that this stage game is repeated *T* times, where *T* is finite or infinite. Each repetition takes place over a period. A typical time (or stage) is denoted by *ί*, where *ί* = *1*, 2, ..., Τ. The interaction evolves as follows [16, 17]:

(1) In period 1, players simultaneously select actions, which we symbolize as *x*^1^=(*x*_1_^1^, *x*_2_^1^,…, *x*_*n*_^1^), where the pointer symbolizes the player and the exponent symbolizes the stage (time period). The action *x*_1_^1^ belongs to the set *X*_*i*_, *i* ∈ *N*. Each player has been informed afterward of the choices of the other players. The performance of player *i* in this period is
  (2) $$\begin{array}{c} u_{i}\left(x^{1}\right),\;i\in N. \end{array}$$
(2) In period 2, players choose at the same time actions *x*^2^=(*x*_1_^2^, *x*_2_^2^,…, *x*_*n*_^2^). Each player has been informed afterward of the choices of the other players. Player's *i* odds are
  (3) $$\begin{array}{c} u_{i}\left(x^{2}\right),\;i\in N. \end{array}$$
(3) In period *T*, players choose actions *x*^*T*^=(*x*_1_^*T*^, *x*_2_^*T*^,…, *x*_*n*_^*T*^). The player's odds are
  (4) $$\begin{array}{c} u_{i}\left(x^{T}\right),\;i\in N. \end{array}$$
(4) If *T* is finite, the interaction is complete (at the end of period T). Otherwise, the game continues in perpetuity

In the repetitive game, we suggest each player earns a sequence of payoffs (one payoff for each period). This yield sequence is assumed to be valued using the discounted sum of the sequence terms. The term discount expresses the assumption that a person does not value current and future returns equally. Parameter *δ* is the discount rate of an individual [18]. The closer *δ* is to 0, the less the individual values a future versus a present performance. In other words, the smaller the *δ* is, the less the person is interested in the future or the more impatient he is. Conversely, the higher the *δ* (the closer it is to 1) is, the more a person values a future performance or is more patient [19–21].

Let a finite terminal story *h*^*T*^=(*x*^1^, *x*^2^,…, *x*^*T*^). If the discount rate of player *i* is given by the parameter *δ*_*i*_ ∈ (0,1), then the discounted sum of the payoffs of player *i* is

(5) $$\begin{array}{c} V_{i}\equiv u_{i}\left(x^{1}\right)+\delta_{i}u_{i}\left(x^{2}\right)+\delta_{i}^{2}u_{i}\left(x^{3}\right)+\cdots +\delta_{i}^{T-1}u_{i}\left(x^{T}\right)=\sum_{t=1}^{T}\delta_{i}^{t-1}u_{i}\left(x^{t}\right). \end{array}$$

Let now an infinite terminal story *h*^*∞*^=(*x*^1^, *x*^2^,…). In this case, the discounted sum of player's payoffs is

(6) $$\begin{array}{c} V_{i}\equiv u_{i}\left(x^{1}\right)+\delta_{i}u_{i}\left(x^{2}\right)+\delta_{i}^{2}u_{i}\left(x^{3}\right)+\cdots =\sum_{t=1}^{\infty }\delta_{i}^{t-1}u_{i}\left(x^{t}\right). \end{array}$$

In the case of infinity *T*, instead of the discounted sum of the odds, we use the discounted average of the player's odds as a valuation function:

(7) $$\begin{array}{c} u_{i}^{t}=u_{i}\left(x^{t}\right),\;t=1,2,\ldots . \end{array}$$

Based on the relation, player *i* obtains the sequence of odds (*u*_*i*_^1^, *u*_*i*_^2^,…). We define a number *c* such that *i* is indifferent between the series of yields (*u*_*i*_^1^, *u*_*i*_^2^,…) and the sequence (*c*, *c*_1_, ..., *c*_*n*_). So, we take the following relation [22]:

(8) $$\begin{array}{c} V_{i}=\sum_{t=1}^{\infty }\delta_{i}^{t-1}u_{i}^{t}\\ =\sum_{t=1}^{\infty }\delta_{i}^{t-1}c. \end{array}$$

However, it is true that

(9) $$\begin{array}{c} \sum_{t=1}^{\infty }\delta_{i}^{t-1}c=\frac{c}{1-\delta_{i}}. \end{array}$$

Consequently, we have

(10) $$\begin{array}{c} V_{i}=\sum_{t=1}^{\infty }\delta_{i}^{t-1}u_{i}^{t}\\ =\frac{c}{1-\delta_{i}}, \end{array}$$

so that

(11) $$\begin{array}{c} \left(1-\delta_{i}\right)V_{i}=\left(1-\delta_{i}\right)\sum_{t=1}^{\infty }\delta_{i}^{t-1}u_{i}^{t}=c. \end{array}$$

The term (1 − *δ*_*i*_)*V*_*i*_ is the discounted average of the yield sequence (*u*_*i*_^1^, *u*_*i*_^2^,…). The functions *V*_*i*_ and (1 − *δ*_*i*_)*V*_*i*_ express the same preferences since one is a positive monotonic transformation. Thus, the set of all terminal stories is the set of all sequences [3, 23]:

(12) $$\begin{array}{c} h^{\infty }=\left(x^{1},x^{2},\ldots \right). \end{array}$$

So, player *i* evaluates the terminal history *h*^*∞*^ based on the function:

(13) $$\begin{array}{c} V_{i}=\sum_{t=1}^{\infty }\delta^{t-1}u_{i}\left(x^{t}\right), \end{array}$$

or it is equivalent:

(14) $$\begin{array}{c} \left(1-\delta \right)V_{i}=\left(1-\delta \right)\sum_{t=1}^{\infty }\delta^{t-1}u_{i}\left(x^{t}\right). \end{array}$$

So, we have the repetitive game *G*^*∞*^(*δ*).

A rule that dictates the player's behavior in response to any and all scenarios is an example of a strategy. Consequently, a single-player strategy is a guideline for picking actions at each stage (repetition) of the stage game as a function of previous decisions when playing a game that features observable actions and is repetitive. To be more exact, the energy that the player will pick at each narrative stage is determined by the strategy used in a game with just one player and repeated steps. The action must be feasible; that is, it must belong to all the available options of the gaming stage [24, 25].

Odds pairs corresponding to the four pairs of clear strategies are feasible (in the sense that there are strategies that generate specific returns). It should also be noted that all convex combinations of two or more yield pairs of pure methods constitute achievable yields (using mixed strategies) [5, 26].

**Figure 1:**
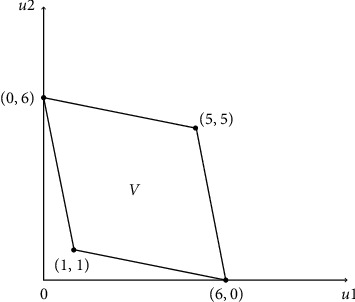
Possible payoffs in the form of a diagram.

In general, the total possible odds of the stage game are given by the quadrilateral in Figure 1, which has as vertices the four pairs of payoffs corresponding to the clear strategies described by the set {(5, 5), (0, 6), (6, 0), (1,1)}.

## 4. Application Testing

In order to model the proposed system, a specialized threat scenario was implemented with an application study in the music industry. This was done because the large number of visitors combined with the enormous amounts of music content spent on a daily basis creates a new landscape of threats, in which the clear strategies for cybersecurity need to be rearranged on an ongoing basis. According to this logic, they frequently employ advanced techniques, including zero-day attacks, to launch attacks on music streaming platforms that modern cybercriminals have targeted [24, 27, 28].

For the implementation of the proposed system, we consider a static game Γ={*N*, (*X*_*i*_, *u*_*i*_)_*i*∈*N*_}, such that

1. (*u*_1_^*∗*^, *u*_2_^*∗*^,…, *u*_*n*_^*∗*^) is the payoff vector of a Nash equilibrium of Γ (the Nash equilibrium is a solution technique for non-cooperative games with two or more participants in game theory. We assume that each player is aware of the equilibrium strategies that the other players would employ and that no player stands to gain by simply changing his strategy [2] if each participant has decided on a strategy and no one has the option of modifying their strategy. On the contrary, the other players maintain their strategy constant; their set of plans and results are the Nash equilibrium.
2. $\left({\hat{v}}_{1},{\hat{v}}_{2},\ldots ,{\hat{v}}_{n}\right)$ is a vector of possible returns of Γ such that ${\hat{v}}_{i}>u_{i}^{*}$ for each *i*.
3. The discount rate *δ* is quite large.

Then, there is a perfect subgame balance for the game *G*^*∞*^(*δ*) with average odds $\left({\hat{v}}_{1},{\hat{v}}_{2},\ldots ,{\hat{v}}_{n}\right)$.

To prove the above scenario, we will assume that the vector $\left({\hat{v}}_{1},{\hat{v}}_{2},\ldots ,{\hat{v}}_{n}\right)$ can be achieved through a vector of pure strategies $\hat{x}=\left({\hat{x}}_{1},{\hat{x}}_{2},\ldots ,{\hat{x}}_{n}\right)$. That is, $u_{i}\left(\hat{x}\right)={\hat{u}}_{i},i\in N$. It should be noted that it is not necessary to assume clean strategies. If this is not the case, vector V is achieved through mixed strategies [5, 10].

Suppose that the equilibrium returns (*u*_1_^*∗*^, *u*_2_^*∗*^,…, *u*_*n*_^*∗*^) of Γ are derived from the strategy vector (*x*_1_^*∗*^, *x*_2_^*∗*^,…, *x*_*n*_^*∗*^).

We consider the following firing strategy:

(1) Stage *i* = 1:
  - player *i* selects an action ${\hat{x}}_{i}$
(2) Stage *i* > 1:
  - If the result in the previous stages 1, 2, ..., t-1 is $\hat{x}$, player *i* selects an action ${\hat{x}}_{i}$
  - In all other cases, player *i* selects an action *x*_*i*_^*∗*^

We will examine the players' motivations for adopting or not the above strategy.

Let us look at player *i* if all other players adopt the firing strategy. We must determine the optimal response of *i* in each period where it has observed a result $\hat{x}$ and its optimal response in each period where it has observed a result different from $\hat{x}$.

It should be recorded the actions that *i* will take if it observes in a period *t* that in the previous period t-1 a result other than $\hat{x}$ was outcome. Player *i* knows that, in period *t*, the other players will choose the actions *x*_−*i*_^*∗*^ and that this will be done in perpetuity (because they follow the firing strategy). Therefore, the optimal reaction of 1 is to choose *x*_*i*_^*∗*^ in perpetuity.

Let us now see what player *i* will do in those periods for which history contains only the result $\hat{x}$. Suppose that, in some of them, *i* chooses to deviate from the energy ${\hat{x}}_{i}$. The optimal deviation, which we denote by *x*_*i*_^*d*^*d*, is what solves the problem.

In particular,

(15) $$\begin{array}{c} \underset{x_{i}}{\max}u_{i}\left(x_{i},{\hat{x}}_{-i}\right). \end{array}$$

The yield of *i* during the deviation period is

(16) $$\begin{array}{c} u_{i}^{d}\equiv u_{i}\left(x_{i}^{d},{\hat{x}}_{-i}\right), \end{array}$$

where it is true that

(17) $$\begin{array}{c} u_{i}^{d}\geq {\hat{u}}_{i}. \end{array}$$

From the next period, the other players will start choosing *x*_−*i*_^*∗*^, after noticing the choice *x*_*i*_^*d*^ of *i*. The punishment period of *i* will therefore begin. Given this, the optimal response of *i* is to select the energy *x*_*i*_^*∗*^. Thus, from the period following the deviation to perpetuity, *i* will have a payoff of *u*_*i*_^*∗*^ per period. Its average discounted return from the breach is, therefore,

(18) $$\begin{array}{c} \left(1-\delta \right)\left(u_{i}^{d}+\delta u_{i}^{*}+\delta^{2}u_{i}^{*}+\ldots \right)=\left(1-\delta \right)u_{i}^{d}+\delta u_{i}^{*}. \end{array}$$

If, on the contrary, *i*, after each story that contains only $\hat{x}$, chooses the action ${\hat{x}}_{i}$; its return will be ${\hat{u}}_{i}$ because after such a story, the other players choose ${\hat{x}}_{-i}$. For each of the following periods, the same result will be repeated, and *i* will continuously gain ${\hat{u}}_{i}$ per period. The average payoff of *i* in this case is

(19) $$\begin{array}{c} \left(1-\delta \right)\left({\hat{u}}_{i}+\delta {\hat{u}}_{i}+\delta^{2}{\hat{u}}_{i}+\ldots \right)=\left(1-\delta \right)\frac{1}{1-\delta }{\hat{u}}_{i}={\hat{u}}_{i}. \end{array}$$

Therefore, player *i* will select ${\hat{x}}_{i}$ after each story that contains only $\hat{x}$ if

(20) $$\begin{array}{c} {\hat{u}}_{i}\geq \left(1-\delta \right)u_{i}^{d}+\delta u_{i}^{*}⟺\delta \left(u_{i}^{d}-u_{i}^{*}\right)\geq u_{i}^{d}-{\hat{u}}_{i}. \end{array}$$

If the following inequality relations are valid,

(21) $$\begin{array}{c} u_{i}^{d}\geq {\hat{u}}_{i}>u_{i}^{*}, \end{array}$$

so it applies

(22) $$\begin{array}{c} \delta \geq \frac{u_{i}^{d}-{\hat{u}}_{i}}{u_{i}^{d}-u_{i}^{*}}. \end{array}$$

And so

(23) $$\begin{array}{c} \frac{u_{i}^{d}-{\hat{u}}_{i}}{u_{i}^{d}-u_{i}^{*}}\leq 1. \end{array}$$

**Figure 2:**
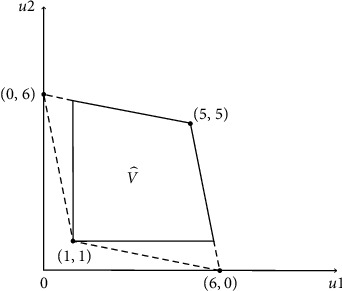
Possible payoffs that exceed Nash payoffs.

The possible payoffs that exceed the Nash payoffs are shown in Figure 2.

Next, to determine which value can best estimate the random variable, we apply Bayesian inference to create a loss function, which will measure how wrong the estimate is, that is, how different the estimation of the parameter is from its actual price. This means that the goal is for the value of the estimate to minimize the loss function. For example, in the absolute error loss function, underestimation and overestimation of the parameter are punished in the same way, depending on the deviation of the estimate. In contrast to the preceding statement, the linear loss function penalizes underestimation with a different weight than overestimation.

The evaluation of the decision rules is done through the risk function, which is defined as the average value of the loss function. If *L*(*δ*, *θ*) is the loss function and *δ* *=* *δ(x)* is the decision rule, the hazard function of the decision rule *δ* is mathematically expressed as

(24) $$\begin{array}{c} R\left(\delta ,\theta \right)=E_{\theta }L\left(\delta ,\theta \right)\\ =\int L\left(\delta \left(x\right),\theta \right)f\left(x|\theta \right)\text{d}x. \end{array}$$

In the Bayesian approach, it is possible to balance the risk function of each decision rule, based on ex-ante personal opinion, through *u(θ)*. So, the Bayesian risk is defined as [29–31]

(25) $$\begin{array}{c} BR\left(\delta \right)=\int_{\Theta }R\left(\delta ,\theta \right)u\left(\theta \right)\text{d}\theta \\ =\int_{\Theta }\int_{X}L\left(\delta \left(x\right),\theta \right)f\left(x|\theta \right)u\left(\theta \right)\text{d}x\text{d}\theta . \end{array}$$

The decision rule that minimizes Bayesian risk is the Bayesian rule. In particular, the Bayesian risk of decision rule *δ*, as a function of the loss of a square error, is equal to

(26) $$\begin{array}{c} E{\left(\delta -\theta \right)}^{2}=\int_{\Theta }\int_{X}{\left(\delta \left(x\right)-\theta \right)}^{2}f\left(x|\theta \right)\text{d}xu\left(\theta \right)\text{d}\theta \\ =\int_{\Theta }\int_{X}{\left(\delta \left(x\right)-\theta \right)}^{2}f\left(x|\theta \right)u\left(\theta \right)\text{d}x\text{d}\theta \\ =\int_{\Theta }\int_{X}{\left(\delta \left(x\right)-\theta \right)}^{2}f\left(x,\theta \right)\text{d}x\text{d}\theta \\ =\int_{X}\int_{\Theta }{\left(\delta \left(x\right)-\theta \right)}^{2}p\left(\theta |x\right)g\left(x\right)\text{d}\theta \text{d}x\\ =\int_{X}\int_{\Theta }{\left(\delta \left(x\right)-\theta \right)}^{2}p\left(\theta |x\right)\text{d}\theta g\left(x\right)\text{d}x. \end{array}$$

Since the margin function g(*x*) is a nonnegative function, we are interested in minimizing the

(27) $$\begin{array}{c} I\left(x\right)=\int_{\Theta }{\left(\delta \left(x\right)-\theta \right)}^{2}p\left(\theta |x\right)\text{d}\theta , \end{array}$$

which becomes

(28) $$\begin{array}{c} I\left(x\right)=\int_{\Theta }\left(\delta {\left(x\right)}^{2}-2\theta \delta \left(x\right)+\theta^{2}\right)p\left(\theta |x\right)\text{d}\theta \\ =\delta {\left(x\right)}^{2}\int_{\Theta }p\left(\theta |x\right)\text{d}\theta -2\delta \left(x\right)\int_{\Theta }\theta p\left(\theta |x\right)\text{d}\theta +\int_{\Theta }\theta^{2}p\left(\theta |x\right)\text{d}\theta \\ =\int_{\Theta }p\left(\theta |x\right)\text{d}\theta \left(\delta {\left(x\right)}^{2}-2\delta \left(x\right)\frac{\int_{\Theta }\theta p\left(\theta |x\right)\text{d}\theta }{\int_{\Theta }p\left(\theta |x\right)\text{d}\theta }\right)+\int_{\Theta }\theta^{2}p\left(\theta |x\right)\text{d}\theta \\ =\int_{\Theta }p\left(\theta |x\right)\text{d}\theta {\left(\delta \left(x\right)-\frac{\int_{\Theta }\theta p\left(\theta |x\right)\text{d}\theta }{\int_{\Theta }p\left(\theta |x\right)\text{d}\theta }\right)}^{2}+\left[\int_{\Theta }\theta^{2}p\left(\theta |x\right)\text{d}\theta -\frac{\left.{\left(\int_{\Theta }\theta p\left(\theta |x\right)\text{d}\theta \right)}^{2}\right]}{\int_{\Theta }p\left(\theta |x\right)\text{d}\theta }\right]. \end{array}$$

The above function is minimized for *δ*(*x*) when

(29) $$\begin{array}{c} \delta \left(x\right)=\frac{\int_{\Theta }\theta p\left(\theta |x\right)\text{d}\theta }{\int_{\Theta }p\left(\theta |x\right)\text{d}\theta }\\ =E\left(\theta |x\right). \end{array}$$

So, through a hypothesis check, we can determine the correctness of a hypothesis that concerns an action without dealing with estimating the possible values of the strategy. Based on this view, it is possible to predict the future prices of the process as, through the ex-post distribution, the forecasting process is immediate and accurate.

Assuming that, for a random sample of observations *x* = *x*_1_, *x*_2_, *…*, *x*_*n*_, it is desirable to predict the future observation *y*, and it is necessary to calculate the a posteriori distribution of prediction *π*(*y*|*x*). For the mathematical calculation of the ex-post distribution, the mathematical analysis of the ex-prediction distribution *g*(*x*) is required:

(30) $$\begin{array}{c} g\left(x\right)=\int_{\theta }f\left(x,\theta \right)\text{d}\theta \\ =\int_{\theta }f\left(x|\theta \right)u\left(\theta \right)\text{d}\theta . \end{array}$$

Observing the equation, it is easy to see that the integral contains the product of the probability function *f*(*x*|*θ*), with the ex-ante distribution *u(θ)*. Thus, based on the above procedure, the mathematical definition of the ex-post prediction distribution *π*(*y*|*x*) will be made:

(31) $$\begin{array}{c} \pi \left(y|x\right)=\int_{\theta }f\left(y,\theta |x\right)\text{d}\theta \\ =\int_{\theta }f\left(y|\theta ,x\right)p\left(\theta |x\right)\text{d}\theta \\ =\int_{\theta }f\left(y|\theta \right)p\left(\theta |x\right)\text{d}\theta . \end{array}$$

The probability function of the following observations is equal to the integral of the joint probability function *f*(*y*, *θ*|*x*). It should be noted that this time the shared distribution function is bound to the observed data. Re-applying the Bayesian theorem, we conclude that the ex-post distribution of prediction is again equal to the product of probability *f*(*y*|*θ*), but this time, with the ex-post distribution *p*(*θ*|*x*), this is completed for the variable *θ*.

The effect of the ex-post information is therefore apparent. It should be noted that, from the probability function *f*(*y*, *θ*|*x*), we came to the probability function if all a posteriori information originates from the parameter *θ*.

To prove the validity of the methodology described above, we will make the assumption that we will count the number of privilege escalation attacks made against a randomly chosen account on the music streaming platform that is the focus of the current example.

Suppose that the number of specific attacks over a period follows the “Poisson” distribution (*θ*), *θ* ∈ Θ= (0, ∞), i.e.,

(32) $$\begin{array}{c} f\left(x_{i}|\theta \right)=e^{-\theta }\frac{\theta^{x_{i}}}{x_{i}!},\;i=1,2,\ldots ,n. \end{array}$$

For *n* observation time, the probability of the sample is

(33) $$\begin{array}{c} L\left(\theta |x\right)=\prod_{i=1}^{n}f\left(x_{ı}|\theta \right)\\ =\prod_{i=1}^{n}e^{-\theta }\frac{\theta^{x_{i}}}{x_{i}!}\\ =e^{-n\theta }\frac{\theta^{\sum x_{i}}}{\prod x_{i}!}. \end{array}$$

Suppose that the ex-ante distribution of *θ* follows the *Gamma* distribution *(a β)*, where *a*, *β* > 0; quantities are known as a function of probability density:

(34) $$\begin{array}{c} u\left(\theta \right)=\frac{\theta^{\alpha -1}e^{-\left(\theta /\beta \right)}}{\beta^{\alpha }\Gamma \left(\alpha \right)}. \end{array}$$

Therefore, the distribution margin is

(35) $$\begin{array}{c} g\left(x\right)=\int_{0}^{\infty }L\left(\theta |x\right)u\left(\theta \right)\text{d}\theta \\ =\int_{0}^{\infty }\left(e^{-n\theta }\frac{\theta^{\sum x_{i}}}{\prod x_{i}!}\right)\left(\frac{\theta^{\alpha -1}e^{-\left(\theta /\beta \right)}}{\beta^{\alpha }\Gamma \left(\alpha \right)}\right)\text{d}\theta \\ =\frac{1}{\beta^{\alpha }\Gamma \left(\alpha \right)\prod x_{i}!}\int_{0}^{\infty }e^{-n\theta -\left(\theta /\beta \right)}\theta^{\sum x_{i}+\alpha -1}\text{d}\theta \\ =\frac{{\left(\beta /n\beta +1\right)}^{\sum x_{i}+\alpha }\Gamma \left(\sum x_{i}+\alpha \right)}{\beta^{\alpha }\Gamma \left(\alpha \right)\prod x_{i}!}\int_{0}^{\infty }\frac{e^{-\theta \left(n\beta +1/\beta \right)}\theta^{\left(\sum x_{i}+\alpha \right)-1}}{{\left(\beta /n\beta +1\right)}^{\sum^{}x_{i}+\alpha }\Gamma \left(\sum x_{i}+\alpha \right)}\text{d}\theta \\ =\frac{{\left(\beta /n\beta +1\right)}^{\sum x_{i}+\alpha }\Gamma \left(\sum x_{i}+\alpha \right)}{\beta^{\alpha }\Gamma \left(\alpha \right)\prod x_{i}!}. \end{array}$$

Therefore, the a posteriori distribution of *θ* is

(36) $$\begin{array}{c} p\left(\theta |x\right)=\frac{L\left(\theta |x\right)u\left(\theta \right)}{g\left(x\right)}\\ =\frac{\left(e^{-n\theta }\left(\theta^{\sum x_{i}}/\prod x_{i}!\right)\right)\left(\theta^{\alpha -1}e^{-\left(\theta /\beta \right)}/\beta^{\alpha }\Gamma \left(\alpha \right)\right)}{g\left(x\right)}\\ =\frac{\left\{e^{-\theta \left(n\beta +1/\beta \right)}\theta^{\sum x_{i}+\alpha -1}/\prod x_{i}!\beta^{\alpha }\Gamma \left(\alpha \right)\right\}}{\left\{{\left(\beta /n\beta +1\right)}^{\sum x_{i}+\alpha }\Gamma \left(\sum x_{i}+\alpha \right)/\beta^{\alpha }\Gamma \left(\alpha \right)\prod x_{i}!\right\}}\\ =\frac{e\left(n\beta +1/\beta \right)\theta^{\left(\sum x_{i}+\alpha \right)-1}}{{\left(\beta /n\beta +1\right)}^{\sum^{}x_{i}+\alpha }\Gamma \left(\sum x_{i}+\alpha \right)}. \end{array}$$

From the above relation, it becomes clear that the a posteriori distribution of *θ* is [32]

(37) $$\begin{array}{c} \text{Gamma}\left(\sum x_{i}+\alpha ,\frac{\beta }{n\beta +1}\right). \end{array}$$

The ex-post distribution following the *Gamma* distribution was expected since the *Gamma* distribution family, where the ex-distribution belongs, is conjugated to the Poisson distribution.

The ex-post average value for the above *Gamma* distribution is [30, 32, 33]

(38) $$\begin{array}{c} E\left(\theta |x\right)=\left(\sum_{i=1}^{n}x_{i}+\alpha \right)\left(\frac{\beta }{n\beta +1}\right)\\ =\frac{\beta \sum x_{i}}{n\beta +1}+\frac{\alpha \beta }{n\beta +1}\\ =\frac{n\beta }{n\beta +1}\bar{x}+\frac{1}{n\beta +1}\alpha \beta . \end{array}$$

Let *r* ~ Poisson(*θ*) be a new independent observation. The equation gives the forecast distribution:

(39) $$\begin{array}{c} \pi \left(y|x\right)=\int_{\Theta }f\left(y|\theta \right)p\left(\theta |x\right)\text{d}\theta , \end{array}$$

where for the specific statistical model it is [34, 35]

(40) $$\begin{array}{c} \pi \left(y|x\right)=\int_{\theta }\left(e^{-\theta }\frac{\theta^{y}}{y!}\right)\left(\frac{e^{-\theta \left(n\beta +1/\beta \right)}\theta^{\left(\sum x_{i}+\alpha \right)-1}}{{\left(\beta /n\beta +1\right)}^{\sum x_{i}+\alpha }\Gamma \left(\sum x_{i}+\alpha \right)}\right)\text{d}\theta \\ =\frac{1}{y!{\left(\beta /n\beta +1\right)}^{\sum x_{i}+\alpha }\Gamma \left(\sum x_{i}+\alpha \right)}\int_{\theta }e^{-\theta \left(n\beta +\beta +1/\beta \right)}\theta^{\left(\sum x_{i}+\alpha +y\right)-1}\text{d}\theta \\ {\left.=\frac{{\left(\beta /n\beta +\beta +1\right)}^{\sum x_{i}+\alpha +y}\Gamma \left(\sum x_{i}+\alpha +y\right)}{{\left(\beta /n\beta +1\right)}^{\sum x_{i}+\alpha }\Gamma \left(\sum x_{i}+\alpha \right)\Gamma \left(y|+|1\right)}\right)}^{y}\\ =\frac{\Gamma \left(\sum x_{i}+\alpha +y\right)}{\Gamma \left(\sum x_{i}+\alpha \right)\Gamma \left(y|+|1\right)}{\left(\frac{n\beta +1}{n\beta +\beta +1}\right)}^{\sum x_{i}+\alpha }\left(\frac{\beta }{n\beta +\beta +1}\right), \end{array}$$

which is a negative binomial with parameters [36–38]:

(41) $$\begin{array}{c} \left(\sum x_{i}+\alpha ,\frac{\beta }{n\beta +\beta +1}\right). \end{array}$$

## 5. Conclusions

This research offered an original and forward-thinking application of game theory and cutting-edge machine learning methods for adaptive cyber protection measures. It suggested a methodology based on game theory, which concerns the study of elements that characterize situations of competitive interdependence with an emphasis on the decision-making process of more than one decision maker. We make the assumption that every player is informed of the equilibrium tactics that the other players will use and that no player has anything to gain by merely altering the strategy that he uses. Therefore, in order to figure out which value can provide the most accurate estimation of the random variable, we use Bayesian inference to devise a loss function. This function will measure how inaccurate the estimate is, or more specifically, how far off the estimation of the parameter is from the actual price. This indicates that the objective is to have the value of the estimate be as low as possible concerning the loss function.

Specifically, the study of features that characterizes conditions of competitive interdependence includes the opponents. To be more specific, a methodology based on a repeated game is used to examine cyber-attacks and model behaviors and research how defenders and attackers make decisions in an environment where they compete. The unique approach developed can forecast the future moves in the game to generate the proper countermeasures and apply the most acceptable cyber defense tactics that govern a company. This ability is based on the use of Bayesian inference, which is a type of statistical inference. The suggested system was tested with great success in a particular application scenario in the digital music sector and how to cope with upcoming cyber-attacks. The testing was successful on both fronts.

A crucial step in further developing the proposed model is the further investigation of how they can adapt the method parameters to processes of modern and asynchronous change of the initial parameters of the evaluators. This is one of the processes that will further the development of the proposed model. As a result, the expansion and empirical exploration of the characteristics of the method estimators in finite samples, which call for the use of Monte Carlo simulations, is also a vital component of the proposed system's evolutionary parameter.

## Data Availability

The data used in this study are available from the author upon reasonable request.
